# Spontaneous Calcium Transients Recorded from Striatal Astrocytes in a Preclinical Model of Autism

**DOI:** 10.1007/s11064-024-04218-5

**Published:** 2024-08-09

**Authors:** Hugo Saavedra-Bonilla, Durairaj Ragu Varman, Daniel Reyes-Haro

**Affiliations:** 1 Departamento de Neurobiología Celular y Molecular, Instituto de Neurobiología, Universidad Nacional Autónoma de México, Campus Juriquilla, Boulevard Juriquilla 3001, Juriquilla, Querétaro CP76230 Mexico; 2 Department of Pharmacology and Toxicology, Virginia Commonwealth University, Richmond, VA 23298 USA

**Keywords:** Autism, Striatum, Glial cells, Valproic acid, GABA, Calcium signaling

## Abstract

Autism spectrum disorder (ASD) is known as a group of neurodevelopmental conditions including stereotyped and repetitive behaviors, besides social and sensorimotor deficits. Anatomical and functional evidence indicates atypical maturation of the striatum. Astrocytes regulate the maturation and plasticity of synaptic circuits, and impaired calcium signaling is associated with repetitive behaviors and atypical social interaction. Spontaneous calcium transients (SCT) recorded in the striatal astrocytes of the rat were investigated in the preclinical model of ASD by prenatal exposure to valproic acid (VPA). Our results showed sensorimotor delay, augmented glial fibrillary acidic protein -a typical intermediate filament protein expressed by astrocytes- and diminished expression of GABA_A_-ρ3 through development, and increased frequency of SCT with a reduced latency that resulted in a diminished amplitude in the VPA model. The convulsant picrotoxin, a GABA_A_ (γ-aminobutyric acid type A) receptor antagonist, reduced the frequency of SCT in both experimental groups but rescued this parameter to control levels in the preclinical ASD model. The amplitude and latency of SCT were decreased by picrotoxin in both experimental groups. Nipecotic acid, a GABA uptake inhibitor, reduced the mean amplitude only for the control group. Nevertheless, nipecotic acid increased the frequency but diminished the latency in both experimental groups. Thus, we conclude that striatal astrocytes exhibit SCT modulated by GABA_A_-mediated signaling, and prenatal exposure to VPA disturbs this tuning.

**Supplementary Information:**

The online version contains supplementary material available at 10.1007/s11064-024-04218-5.

## Introduction

Autism spectrum disorder (ASD) is characterized by social and sensorimotor impairments, with repetitive patterns of interest and behaviors. On the other hand, valproic acid (VPA) is an antiepileptic medication also effective for bipolar disorder. However, administration of VPA during pregnancy increases the risk of ASD in offspring, and a single prenatal dose is used as a preclinical model [1, 2]. Motor stereotypies and repetitive behaviors observed in ASD are commonly associated to the dorsolateral striatum, while disordered aggregation of striosomal cells reported in the VPA model could potentially be related to social and sensorimotor impairments [3, 4]. The cellular organization of the striatum includes medium spiny neurons (MSNs; 95%) and interneurons (cholinergic and GABAergic). The expression of dopamine receptors D1 or D2 identifies MSNs of the direct or indirect pathways, respectively [5]. Communication between MSNs (D1 and D2) and astrocytes is circuit specific [6]. Interestingly, MSNs showed reduced tonic inhibition when GABA transporter 3 (GAT-3) expression was increased by calcium transient attenuation in astrocytes, affecting the striatal microcircuitry in vivo and leading to self-grooming behavior, associated with human obsessive-compulsive disorder [7]. Calcium release from intracellular stores requires activation of type 2 inositol 1,4,5-trisphosphate receptors (IP_3_R_2_) in astrocytes, and gene mutations of this receptor are linked to ASD. Accordingly, conditional knockout of the astrocytic IP_3_R_2_ in the prefrontal cortex resulted in reduced purinergic gliotransmission with abnormal social interaction and repetitive behavior [8]. Overall, these data strongly suggest that calcium signaling in astrocytes may be perturbed by ASD. Thus, we studied SCT and modulation by GABA_A_ receptors is striatal astrocytes, and tested if this signaling is disturbed in mice prenatally exposed to VPA.

## Materials and Methods

### Animals

Mice were handled according to the National Institute of Health’s Guide for the Care and Use of Laboratory Animals and the Institutional Committee for the Care and Use of Laboratory Animals of Instituto de Neurobiología - UNAM. Briefly, pregnancy of Wistar rats or the transgenic mouse line GFAP-eGFP [9] were confirmed by a vaginal plug at embryonic day 0 (E0). The pregnant rodents were housed individually under controlled temperature and light/dark cycle (12/12 h); food and water were available *ad libitum*. Sterilized saline solution (0.9%) or VPA (Sigma-Aldrich, St. Louis, MO, U.S.) were administered in a single intraperitoneal injection on E12.5 for Wistar rats (500 mg/Kg) [10]. Only male pups were selected based on ASD incidence (4:1) [11–13]. At least three litters were used for each experimental group and animals (N) for histological and behavioral studies, while the number of slices (n) is referred for calcium imaging experiments.

### Sensorimotor Testing

Sensorimotor impairments in pups (P8) were identified with a prognostic behavioral test that estimated the latency to reach the nest for control (CTL) and VPA groups (N_ctl_ = 54, N_vpa_ = 68) as previously described [10]. The experiment ended once the head of the mouse touched the home bedding [1, 2, 10].

### Western Blot Analysis

Five independent sets of protein samples isolated from the striatum were collected in five different stages of development (E16 to P30): E16 (N_ctl_=8; N_vpa_=8), P4 (N_ctl_=12; N_vpa_=12), P8 (N_ctl_=16; N_vpa_=16), P18 (N_ctl_=16; N_vpa_=16), P30 (N_ctl_=14; N_vpa_=16). Briefly, the tissue was homogenized in iced-cold glycine lysis buffer (in mM: 300 sucrose, 200 Glycine, 150 NaCl, 50 EDTA, 50 EGTA, pH 9.0) and protease inhibitor (Sigma-Aldrich, St. Louis, MO, U.S.), followed by isolation and quantification of the protein with a Bradford assay (Bio-Rad, Hercules, CA, U.S.) [14]. The protein (10 µg per lane) was resolved in a 10% polyacrylamide gel, transferred to PVDF membranes, blocked with 5% non-fat dry milk in Tris-buffered saline (TBS), 0.1% Tween 20 (TBS-T) for 3 h at room temperature. The membranes were incubated with the primary antibody goat polyclonal anti-GFAP 1:1,000 (Santa Cruz, Dallas, TX, U.S.), rabbit polyclonal anti- GABA_A_-ρ3 1:1,000 (Santa Cruz, Dallas, TX, U.S.) or rabbit anti-Actin 1:1,000 (Santa Cruz, Dallas, TX, U.S.) overnight at 4 °C. The membranes were rinsed with TBS-T (3 × 15 min each) and incubated (3 h) with the corresponding antibodies (rabbit anti-goat IgG-AP or goat anti-rabbit IgGAP; 1:2,000) (Santa Cruz, Dallas, TX, U.S.), washed again with TBS-T (15 min, 3 x) and detected through alkaline phosphatase activity with the BCIP/NBT AP-conjugate substrate reaction kit (Bio-Rad, Hercules, CA, U.S.). Western blot images were acquired with the image-based Gel Doc^™^ EZ Gel Documentation System (Bio-Rad, Hercules, CA, U.S.). Image Lab 3.0 software (BioRad, Hercules, CA, U.S.) was used for optical density estimation and normalized with the β-Actin bands.

### Staining and Functional Identification Of Astrocytes

Sulforhodamine 101 (SR101), a red fluorescent xanthene derivative is widely used for astrocyte identification. SR101 uptake was observed in 92% of the GFAP-EGFP+ cells and approximately 1/3 of the SR101+ cells were GFAP-EGFP+ (data not shown). Astrocytes uptake SR101 through the thyroid hormone transporter OATP1C1 [15]. Thus, slices were incubated for 20 min with 1 µM of sulforhodamine 101 (SR101) added to the artificial cerebrospinal fluid (aCSF) at 37 °C. The slices were rinsed for 10 min [15, 16]. Striatal astrocytes were identified by SR101 staining in coronal slices, and 94% of the SR101 + cells uploaded the calcium indicator Fluo-4AM (378/404 cells, *n* = 12, *N* = 6). Astrocytes express purinergic receptors and functional response to ATP results in evoked calcium transients [17]. Thus, extracellular application of ATP (100 µM) on striatal slices evoked intracellular calcium transients in 88% of SR101 + cells (332/378 cells, *n* = 12, *N* = 6) (Suppl. Figure 1). We conclude that SR101 is a confident tool for identification of striatal astrocytes.

### Brain Slices and Calcium Imaging

The protocol was described before [18–20]. Briefly, coronal slices (250 μm) containing the striatum were obtained from Wistar rats (P8-P10) with a vibratome (VT1000s, Leica) and transferred to ice-cold oxygenated aCSF (in mM: 134 NaCl, 26 NaHCO_3_, 10 glucose, 2.5 KCl, 2 CaCl_2_, 1.3 MgCl_2_, 1.25 K_2_HPO_4_, pH = 7.4). The slices were recovered (30 min at least), in oxygenated aCSF and incubated further (30–40 min, at 37 °C) with Fluo-4 AM (10 µM, AAT Bioquest, Sunnyvale, CA, U.S.). The slices were rinsed for 30 min with aCSF, transferred to the recording chamber and perfused with oxygenated aCSF (2 ml/min) at room temperature (20–22 °C). Calcium imaging experiments were performed under a cooled camera (SensiCam; PCO.Edge 4.2, Kelheim, Germany) coupled to an Olympus upright microscope (BX51WI, Miami, FL, U.S.) and X-Cite X-LED1 module (Lumen Dynamics Fremont, CA, U.S.) [18, 19]. Additionally, Picrotoxin (PTX; 50 µM) or Nipecotic acid (NIP; 100 µM) were added to the aCSF preincubated (∼2 min) and perfused during calcium imaging recordings. Astrocytes located in the dorsal striatum were selected for imaging. Briefly, individual cells were selected as regions of interests. The protocol for image acquisition was for 300s at 1 Hz with a X-Cite XLED1 module (Excelitas Technologies). ImageJ/FIJI and OriginPro 8 software were used for image processing and data analysis [20–22]. SCT were only considered for analysis when they were greater than twice the standard deviation of basal noise. SCT are reported as relative changes in fluorescence (ΔF/F). SCT kinetics were analyzed in ClampFit10.4 (Molecular Devices) and OriginPro 8 (Origin Labs. Northampton, MA.U.S.).

### Statistical Analysis

Graphs and statistical analysis were performed with OriginPro 8 and GraphPad Prism software. The analysis included the Shapiro-Wilk test for normal distribution. Data are mean ± S.E.M. Parametric tests were applied to data sets with normal distribution, otherwise non-parametric tests were used. Student’s two-tailed t-tests (paired and unpaired) and two-tailed Mann–Whitney tests, with significances at *P* < 0.05, were applied for most statistical analyses. One-way or two-way ANOVA tests were used for data sets with more than two conditions, followed by Bonferroni *post-hoc* tests.

## Results

### Sensorimotor Delay and Increased Expression of Striatal GFAP

**Fig. 1:**
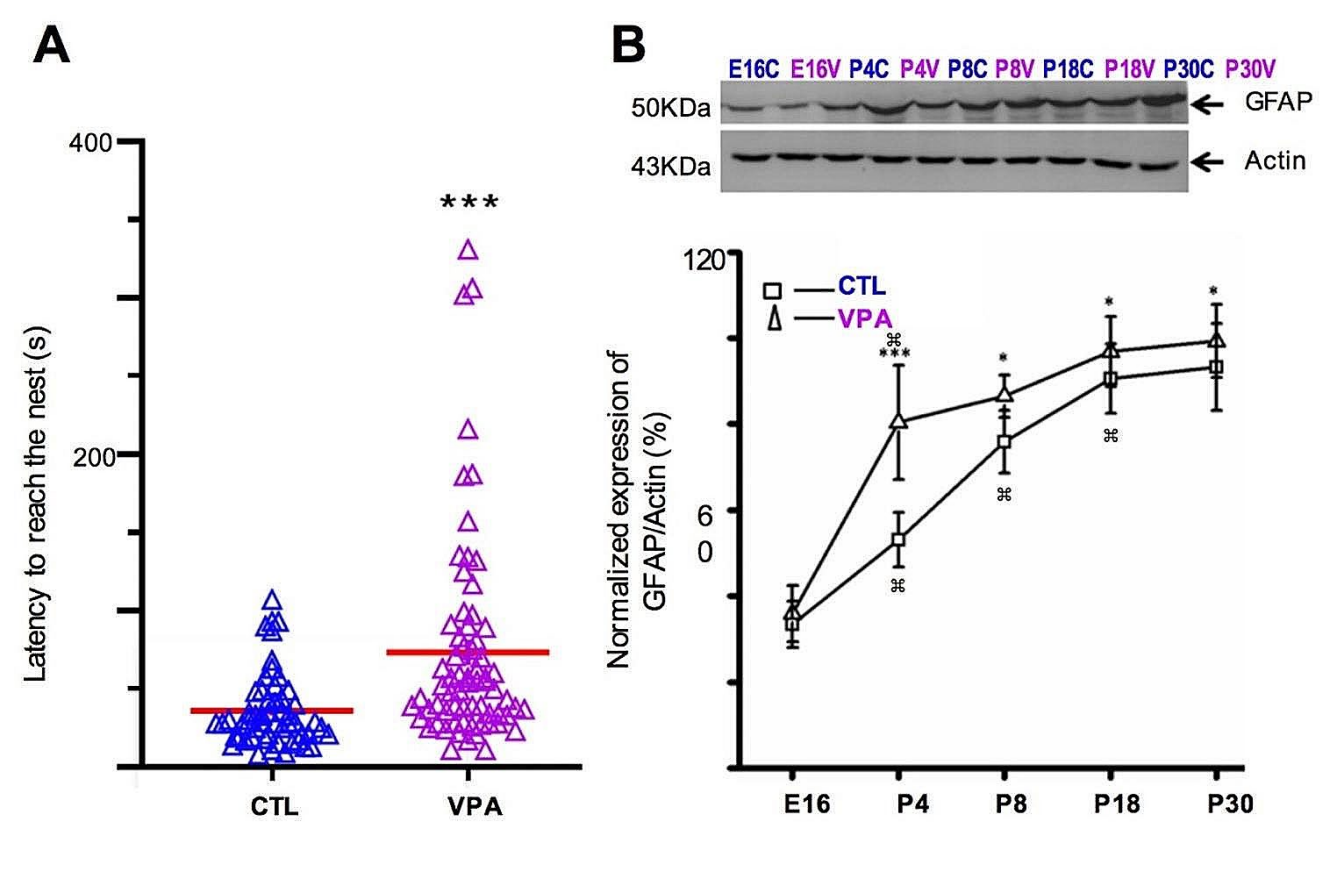
Sensorimotor deficits and increased GFAP expression in the VPA model. (**A**) The latency to reach the nest was significantly augmented by VPA (N_vpa_ = 68 from) when compared to CTL (N_ctl_ = 54) at P8. **(B)** Western blot experiments showed that prenatal exposure to VPA resulted in augmented expression of GFAP through development (from E16 to P30). Data were collected from at least three different litters for each developmental stage, in each group (CTL and VPA). Statistical tests used were Shapiro-Wilk and Mann-Whitney (**A**) and two-way ANOVA followed by a Bonferroni test (**B**). Data are expressed as mean ± S.E.M., *p < 0.05, **p < 0.01, ***p < 0.001

The sensorimotor performance of mice was tested for both experimental groups (CTL and VPA). The latency to reach the nest showed a significant increase in the VPA group (73.2 ± 8.2 s, N_vpa_ = 68, *p* = 0.0002) when compared to CTL (35.9 ± 3.1 s, N_ctl_ = 54) (Fig. 1A).

Western blot studies showed that GFAP expression was significantly augmented by VPA through development (from E16 to P30). The peak of GFAP expression was observed on P4 (+ 30%) (Fig. 1B). We conclude that prenatal exposure to VPA consistently increases GFAP expression through the postnatal development of the striatum.

### Prenatal Exposure to VPA Increases the Frequency of Spontaneous Calcium Transients

**Fig. 2:**
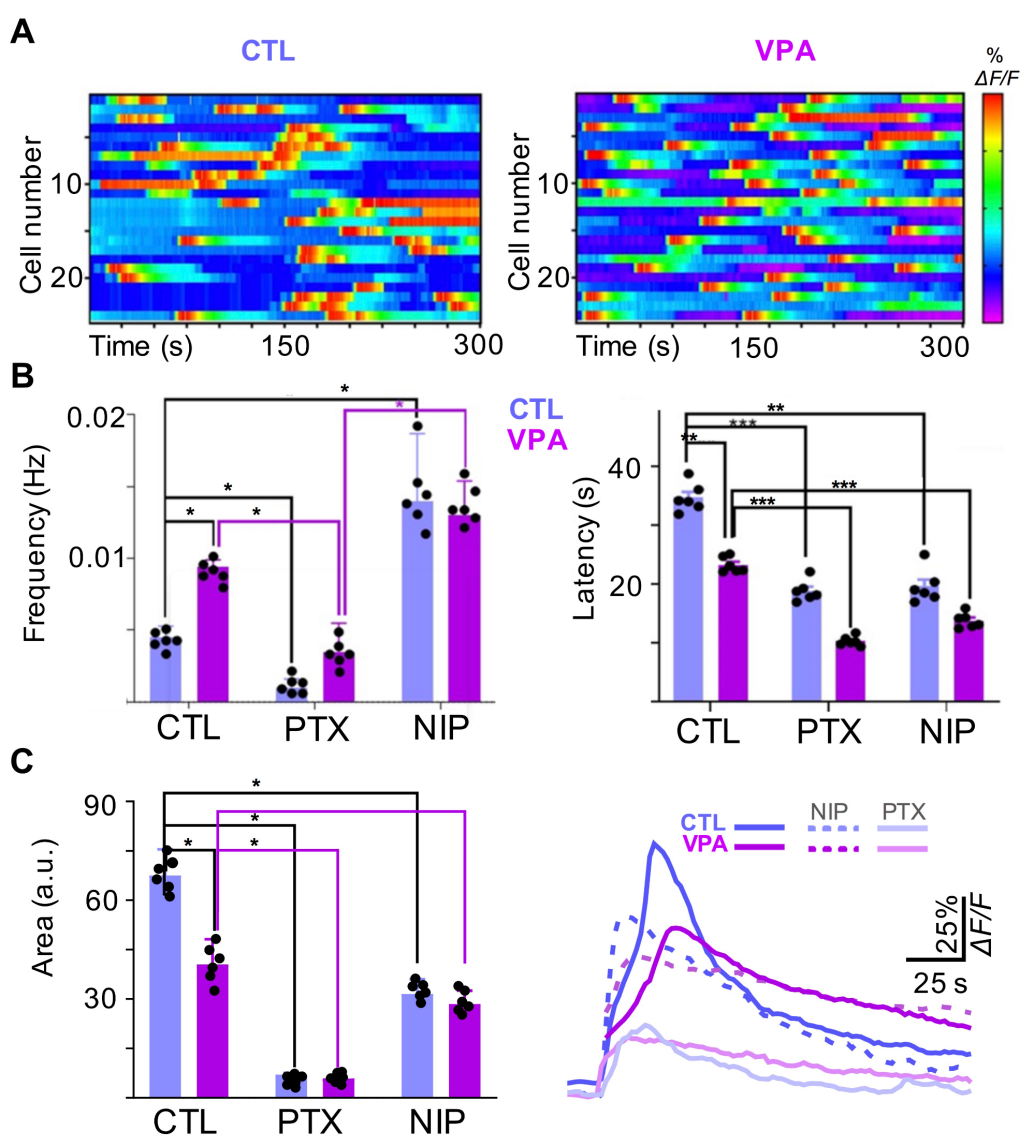
Spontaneous calcium transients (SCT) are modulated by GABA_A_ receptors, and the frequency is increased by VPA. (**A**) Heatmaps showing the temporal course (300 s) of SCT recorded in striatal cells from CTL (left) and VPA (right) experimental groups **(B)** Summary of the mean frequency and latency recorded in CTL (blue bars) and VPA (magenta bars) groups. The effect of picrotoxin (PTX, 50 µM; a non-competitive antagonist of GABA_A_ receptors) and nipecotic acid (NIP, 100 µM; a GABA uptake inhibitor) was tested for both experimental groups. **(C)** Summary of the mean amplitudes corresponding to CTL (blue) and VPA (magenta) groups. Overall, PTX (CTL: 194 cells, n_ctl_=6, N_ctl_= 6; VPA: 172 cells, n_vpa_=6, N_vpa_= 6) and NIP (CTL: 200 cells, n_ctl_=6, N_ctl_=6; VPA: 188 cells, n_vpa_=6, N_vpa_=6) reduced the mean amplitude in CTL and VPA groups. Data analyzed by Shapiro-Wilk, Mann-Whitney U, *p < 0.05, **p < 0.01, ***p < 0.001. Values are mean ± S.E.M

SCT were recorded in striatal astrocytes from rat brain slices (P8-P10). The heat map revealed an increased frequency of SCT in the VPA group (0.0094+/- 0.0005 Hz; from 1067 cells, n_vpa_=34, N_vpa_=12) when compared to CTL (0.0045+/- 0.0008 Hz; from 1212 cells (n_ctl_=35; N_ctl_=12). In contrast, latency of the SCT was reduced by VPA (23 +/- 2 s; from 1067 cells) when compared to CTL (34 +/- 3 s; from 1212 cells) (Fig. 2A, B). Overall, SCT amplitudes were reduced by VPA (-39%; 27 ± 5 a.u., *p* = 0.01 from 172 cells, n_vpa_=6, N_vpa_=6) when compared to CTL (45 ± 5 a.u. from 194 cells, n_ctl_=6, N_ctl_=6).

### GABA_A_ Receptors Modulate Spontaneous Calcium Transients

GABAergic transmission is detected by striatal astrocytes, and we tested whether GABA_A_ receptors modulate SCT. The GABA_A_ antagonist PTX (50 µM) reduced the frequency (Control: -77% and VPA: -63%), latency (Control: -47% and VPA: -55%) and amplitude (Control: -91% and VPA: -89%) of SCT (CTL: 194 cells; n_ctl_=6; N_ctl_= 6; VPA: 172 cells; n_vpa_=6, N_vpa_= 6; *p* = 0.0001) (Fig. 2B, C).

We also tested NIP (100 µM), a GABA uptake inhibitor, to increase the extracellular concentration of endogenous GABA. A significant increase in the frequency (+ 300%), but a decreased latency (-41%), of SCT was observed in striatal astrocytes (CTL: 194 cells; n_ctl_=6; N_ctl_= 6). Similarly, NIP promoted a further rise in the mean frequency of SCT (+ 33%), although the mean latency and amplitude were reduced (-36% and − 30%, respectively) in the VPA group (VPA: 172 cells, n_vpa_=6, N_vpa_= 6; p ˂ 0.01) (Fig. 2B, C).

### Expression of GABA_A_-ρ3 Subunit is Diminished by VPA

**Fig. 3:**
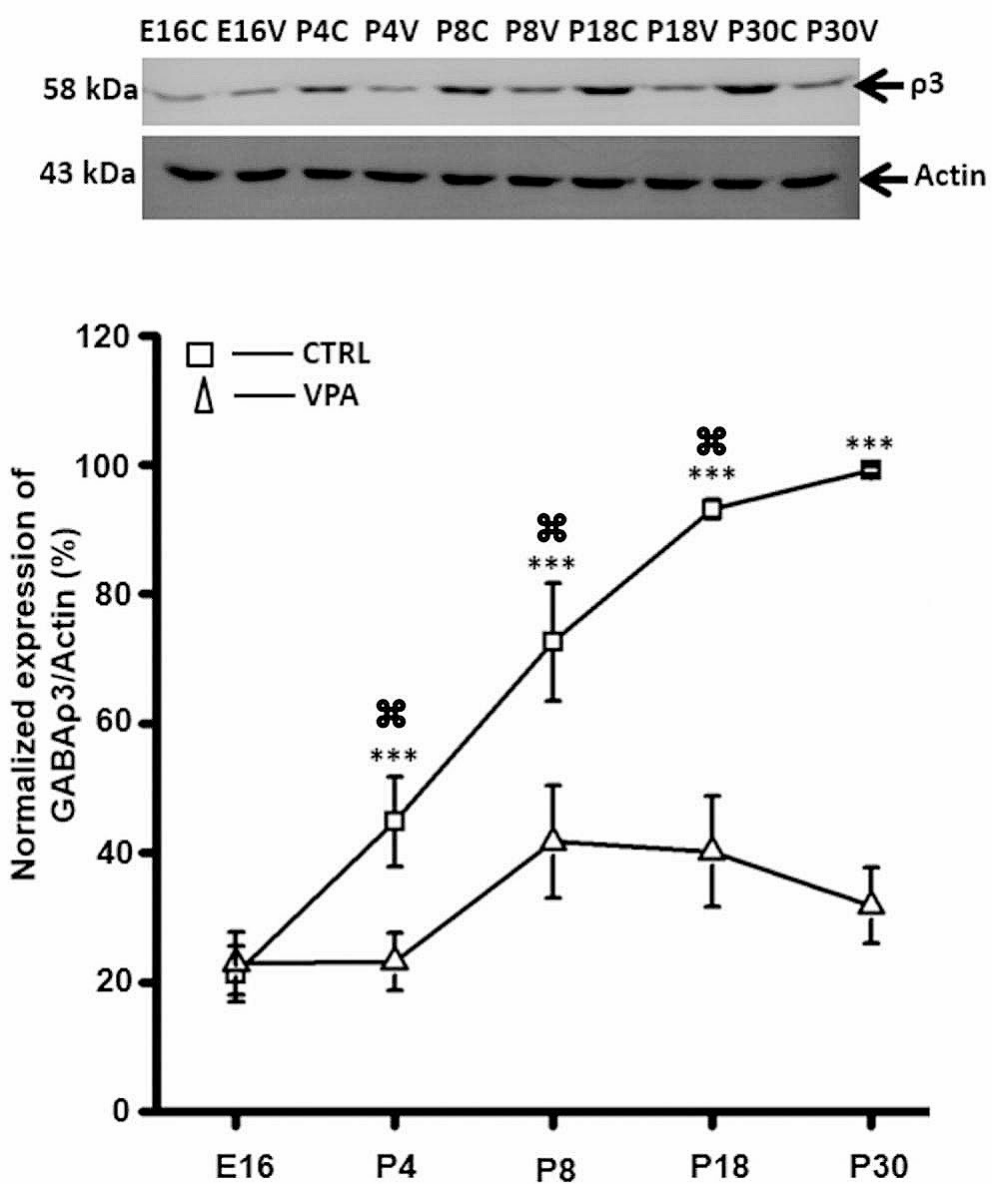
GABA_A_-ρ3 expression is reduced by prenatal exposure to VPA. Western blot experiments showed that GABA_A_-ρ3 expression increased linearly, while it was significantly reduced by VPA through development (from E16 to P30). Data were collected from at least three different litters for each developmental stage, in each group (CTL and VPA). Statistical tests used were two-way ANOVA followed by a Bonferroni test, ***p < 0.001. Data are expressed as mean ± S.E.M

GABA_A_-ρ3 expression was investigated by western blot studies and a significant reduction was observed through postnatal development of the striatum (Fig. 3). The expression of GABA_A_-ρ3 increased linearly through development reaching its peak at P30. However, this pattern was significantly diminished in the VPA model. Our conclusion is that GABAergic signaling through cells expressing GABA_A_-ρ3 is reduced by VPA.

## Discussion

The dorsolateral striatum is linked to motor stereotypies in mammals, and astrocytes are involved in synapse structure and function [23–25]. VPA inhibits histone deacetylase promoting abnormal gene expression and proliferation of astrocytes [26], affecting function and maturation of synaptic circuits through neurodevelopment. Accordingly, sustained increase of GFAP expression was observed through postnatal development of the striatum and correlated with sensorimotor deficits. In agreement, reduced corticostriosomal synapses of striosomal neurons and abnormal ultrasonic vocalizations were seen in this preclinical model of ASD [4]. On the other hand, GABAergic transmission is essential for striatal function, and astrocytes express GABA_A_ receptors [19, 27], but little is known about their role in ASD. In this study we observed sensorimotor deficits, increased expression of GFAP, augmented frequency of SCT recorded in astrocytes, and reduced expression of GABA_A_-ρ3, supporting a disturbed GABA_A_-mediated signaling in the VPA model.

### Sensorimotor Delay and Increased Expression of GFAP

The severity of ASD has been associated with sensorimotor impairments, and experimental data support earlier examinations of motor coordination and sensory responsivity [28–30]. The VPA model provides robust behavioral evidence linked to ASD [1, 2, 31–34]. Moreover, we recently tested behavioral tasks related to the striatal function such as motor skill abilities, repetitive behaviors, and flexibility to shift habits in the VPA model [35]. Briefly, enhanced motor skill learning (rotarod test) was maintained for at least 15 days. Repetitive and compulsive like behaviors were also evaluated through the marble burying test, but no changes were observed. However, a post-hoc evaluation showed a significant increase of digging episodes in the VPA group, indicating a repetitive motor behavior [35]. The aquatic Y-maze has been used to study autistic features by showing an impairment in the reversal learning test. The dorsolateral striatum is known to be involved in reversal learning and a delayed time to get the correct choice in the aquatic Y-maze test was reported, suggesting impaired decision-making [35]. In this study we used the nest-seeking test as a prognostic tool to identify sensorimotor deficits [10, 20]. Our results showed early sensorimotor delay in the VPA group on P8. Thus, this preclinical model reproduces sensorimotor deficits observed in children diagnosed with ASD [36]. On the other hand, impaired aggregation of striosomal cells into clusters was observed during earlier postnatal development in the VPA model [4]. Astrocytes are a key element for maturation and plasticity of synaptic circuits, and we observed an increased expression of GFAP through neurodevelopment of the striatum. Accordingly, the expression of GFAP also increased in the cortex, hippocampus, and cerebellum of the murine model of VPA [20, 26]. The VPA model mimics augmented expression of GFAP reported in brains of postmortem patients diagnosed with ASD [37–39]. These results may reflect a sustained pro-inflammatory environment, but further investigation is required. Overall, social and sensorimotor deficits observed correlate with increased expression of GFAP in the VPA model. Thus, astrocytes are another element to consider in ASD.

### Calcium Signaling in Striatal Astrocytes is Impaired by VPA

Astrocyte activation is linked to intracellular calcium transients mediated by IP_3_R_2_, and mutations of ITPR2 (the corresponding gene) are correlated with ASD. Accordingly, repetitive and reduced social behaviors were reported after conditional knockout of astrocytic-specific IP_3_R_2_ (cKO- IP_3_R_2_) in the medial prefrontal cortex of the mouse [8]. The striatum receives cortical inputs, and we recorded SCT in striatal astrocytes, observing an increased frequency but reduced latency and amplitude in the VPA model. Our results support previous findings in which calcium signaling was reduced in astrocytes, after microinjection of adeno-associated viruses with an astrocyte-specific GfaABC_1_D promoter containing the plasma membrane calcium pump 2 (PMCA2: favors calcium extrusion). The diminished calcium signal in astrocytes (-70%) resulted in augmented grooming, an innate behavior controlled by the dorsal striatum and associated with obsessive-compulsive disorder [7]. Overall, our results show that the calcium code recorded in striatal astrocytes is disturbed in the preclinical model of autism induced by prenatal exposure to VPA.

### GABAergic Signaling is Dysregulated in the VPA Model

The dorsal striatum is involved in self-grooming, and increasing GABA pharmacologically decreases this behavior [40]. Astrocytes regulate striatal MSNs via ambient GABA-mediated neuromodulation, and the GABA transporter 1 (GAT-1) regulates tonic inhibition in the synaptic circuits of the striatum, although GAT-3 becomes relevant when calcium transients are reduced, resulting in increased grooming [7]. We tested NIP, a GABA uptake inhibitor, on SCT recorded in striatal astrocytes. NIP augmented the frequency of SCT in CTL but without effect on VPA group. In contrast, the latency and amplitude of SCT were significantly reduced in both experimental groups. Altogether, our results suggest that NIP increases GABAergic signaling and the frequency of SCT recorded in striatal astrocytes (CTL), similarly to what is observed in the VPA group. The VPA model is a strongly validated preclinical model of ASD in which several GABAergic genes are downregulated in the cortex: GAD65 (-21%), GAD67 (-77%), GABRA1 (-54%), GABRA5 (-57%), and GABRB2 (-55%) [41]. Accordingly, GABAergic signaling dysfunction is related to ASD, and previous studies reported downregulation of several GABA_A_ subunits in postmortem studies of ASD patients [42, 43]. Polymorphisms of some GABA_A_ receptor subunits located on chromosome 15q11-q13 (e.g., GABRB3, GABRA5 and GABRG3) are also implicated in ASD [44]. We tested whether GABA_A_ receptors modulate SCT in striatal astrocytes and observed that PTX blocked ≥ 90% of the spontaneous events. Interestingly, the frequency of SCT, but not the amplitude or the latency, was recovered by PTX to control levels in the VPA model. Our results suggest that SCT recorded in striatal astrocytes are modulated by GABAergic signaling through GABA_A_ receptors. This conclusion is supported by whole-cell patch-clamp recordings demonstrating functional expression of GABA_A_ receptors, and immunofluorescence studies showing GABA_A_-ρ3 expression by ∼ 70% of the GFAP + cells in the striatum of the mouse [19]. Accordingly, western blot studies showed that GABA_A_-ρ3 expression is significantly diminished through postnatal development of the striatum in the VPA model. Moreover, other murine models of ASD have shown GABA_A_ receptor involvement. For example, GABRB3 gene mutations resulted in a reduced tonic but augmented phasic inhibition associated with altered dendrite spine structure, resulting in repetitive behaviors, and diminished social interaction [45]. Additionally, the expression of GABA_A_ subunits is diminished in the murine model of Rett syndrome (MeCP2+/- mouse brain) [46, 47] and the model of Fragile X syndrome (FMR1 knock-out mouse: α3, α4, β1, β2, γ1, γ2) [48–50]. Astrocytes are structural elements of the tripartite synapse and differentially regulate glutamatergic and GABAergic neurons, but the excitatory/inhibitory balance is disturbed by VPA [33, 51]. A recent study showed that co-culture of GABAergic neurons with astrocytes exposed to VPA resulted in a reduced frequency of miniature inhibitory postsynaptic currents (mIPSCs), suggesting a diminished function of the presynaptic terminal. Immunochemical studies confirmed this finding by showing a reduced number of vesicular GABA transporters [52]. Thus, the formation of GABAergic synapses and synaptic neurotransmission are also impaired by VPA-exposed astrocytes, thereby inducing an imbalance in neuronal signaling. Furthermore, VPA exposure promotes gliogenesis and augmented expression of GFAP [26], resulting in morphological hypertrophy and increased number of GFAP + cells in the internal granular layer of the mouse cerebellum [20]. In agreement with these studies, we also observed that prenatal exposure to VPA increased the expression of GFAP, but expression of GABA_A_-ρ3 was diminished through postnatal development of the striatum. These results support the abnormal GABAergic signaling recorded in striatal astrocytes.

### Limitations, Future Directions and Conclusion

Neurodevelopmental disorders like ASD are usually investigated in adult animals and limited studies explore what happen at early stages of postnatal development. A technical limitation for in vivo experiments (for example at P8) is the size and weight of the miniscopes. Thus, we overcome this difficulty by performing calcium imaging studies with coronal slices of the brain containing the striatum (ex-vivo experiments), however, a direct correlation of the results with the sensorimotor test cannot be establish. Future directions should consider chemogenetic manipulation of astrocytes to test the impact on sensorimotor deficits observed in murine models of ASD. Overall, we conclude that sensorimotor deficits, increased expression of GFAP, augmented frequency of SCT recorded in astrocytes, and reduced expression of GABA_A_-ρ3 support a disturbed GABA_A_-mediated signaling in the VPA model.

## Electronic Supplementary Material

Below is the link to the electronic supplementary material.

Supplementary Material 1

## Data Availability

No datasets were generated or analysed during the current study.
