# Supplementary material for: Spontaneous Calcium Transients Recorded from Striatal Astrocytes in a Preclinical Model of Autism
Source: Neurochem Res. 2024 Aug 9;49(11):3069–77. doi: 10.1007/s11064-024-04218-5 (PMC11450070; doi:10.1007/s11064-024-04218-5)
Supplement: Supplementary file 1 — Supplementary Material 1 [file 11064_2024_4218_MOESM1_ESM.docx]

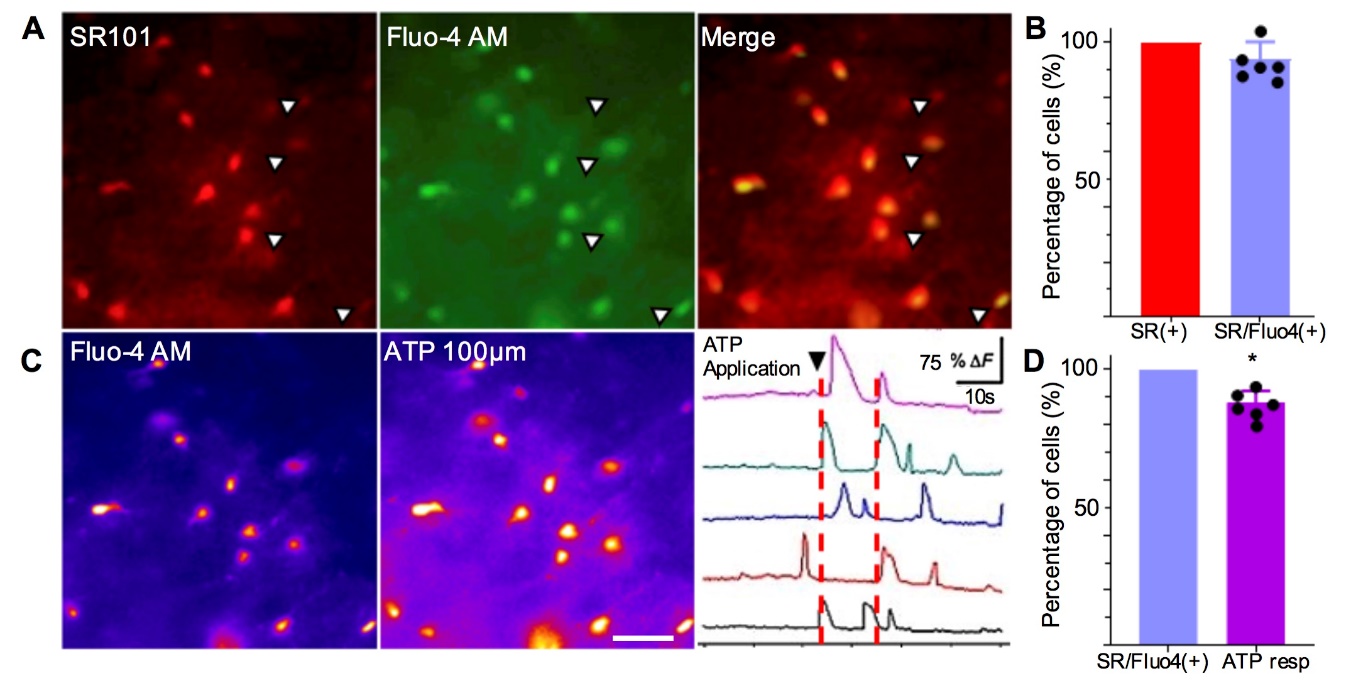


***Suppl. Fig. 1. Identification of striatal astrocytes with sulforhodamine 101 (SR101) labeling.*** ***(A)*** *Representative brain slice containing the dorsal striatum stained with SR101 after preloading with Fluo-4AM. Merge of both images shows co-localization of SR101+ cells with Fluo-4AM. Scale bar: 50 µm.* ***(B)*** *Summary of SR101+ and SR101+/Fluo-4AM cells. The majority of SR101+ cells uploaded Fluo-4AM (94%; 378/404 cells, n=12, N=6).* ***(C)*** *Cells pre-loaded with Fluo-4AM (left image) showed calcium transients evoked by ATP (100 uM) (right image). Five representative cells (1-5) were selected to illustrate calcium transients evoked by ATP.* ***(D)*** *Summary of SR101+/Fluo-4AM cells show that 88% responded to ATP (312/355 cells, n=12, N=6). Data analyzed by one-way ANOVA (B, p=0.074; D, *p=0.02). Values are mean ± S.E.M.*
